# Role of percutaneous closed needle pleural biopsy among patients of undiagnosed exudative pleural effusion

**DOI:** 10.4103/0970-2113.80319

**Published:** 2011

**Authors:** H. S. Hira, Rajiv Ranjan

**Affiliations:** *Department of Pulmonary and Internal Medicine, Maulana Azad Medical College and Lok Nayak Hospital, New Delhi - 110 002, India*

**Keywords:** Closed needle pleural biopsy, pleural biopsy, undiagnosed pleural effusion

## Abstract

**Background:**

Sometimes etiological diagnosis of pleural effusion is difficult despite cytological, biochemical and microbiological tests and labeled as undiagnosed exudative pleural effusions.Aim of present study was to make an etiological diagnosis of pleural effusion.

**Materials and Methods:**

Study group included patients of exudative pleural effusion where etiological diagnosis could not be yielded by conventional cytological, biochemical and microbiological investigations. Pleural tissue was obtained by Cope’s pleural biopsy needle and or thoracoscopy. Pleural biopsy was subjected to histopathology, ZN staining and culture to find the mycobacterium tuberculosis.

**Results:**

Out of 25 patients, 17 (68%) and 8 (32%) were male and female, respectively. Age ranged from 15 to 65 years (mean 31.72). Mean value of serum and pleural fluid LDH was 170.56 U/L and 1080.28 U/L, respectively. Histopathology of 9 (36%) showed epitheloid granuloma with caseation necrosis. In other 9 (36%) patients, epitheloid granulomas (with or without giant cells) was reported. In 5 (20%) patients, histopathology report was of nonspecific chronic inflammation. Histopathology was reported as normal in one case; it turned out to be a case of malignancy. In two (8%) patients, pleural tissue obtained was inadequate for opinions; however, other tests revealed malignancy in one and tuberculosis in other. Ziehl-Neelsen (ZN) stain was positive for AFB in two patients and culture of pleural tissue showed presence of Mycobacterium tuberculosis in three patients.

**Conclusions:**

The role of percutaneous closed needle biopsy of pleura among patients of undiagnosed exudative pleural effusion is still accepted as a diagnostic tool, as this may lead to a specific diagnosis among 76% of cases. This is of particular importance in a developing country like India where the facilities of thoracoscopy and imaging guided cutting needle biopsies are not easily available.

## INTRODUCTION

The etiological diagnosis of exudative effusion is essential. As many as 15% to 20% of all pleural effusions remain undiagnosed despite intensive efforts.[1] In a developing country like India, infections particularly tuberculosis is still the predominant cause.[2,3] More than 40% of patients with an undiagnosed pleural effusion that were followed without treatment developed tuberculosis within 7 years; this study suggested that tuberculosis should be a strong consideration in the diagnosis of undiagnosed pleural effusion.[4] In majority of patients, the diagnosis is apparent by history, physical examination and investigations of pleural fluid. In those, where reaching the diagnosis has failed, the help of invasive diagnostic modalities is required. One of these modalities is percutaneous needle biopsy of parietal pleura. By closed pleural biopsy, 49.1% of undiagnosed exudative pleural effusions could be diagnosed.[5] Closed pleural biopsy provides the highest diagnostic yield in cases of pleural tuberculosis and malignancy, the two most important causes of exudative pleural effusion.[6]

Needle biopsy of pleura was first described in 1955 using Vim Silverman needle[7] and later Abram,[8] Cope[9] and Raja[10] introduced different types of needle and were known by the inventors’ name. Needle-like Tru-cut have been used occasionally.[11] Biopsy from visceral pleura had also been taken successfully and shown higher yield in diagnosis.[12]

Surgical procedures like thoracoscopy and thoracotomy may help to obtain the pleural tissue. Lately flexible thoracoscopy using local anesthesia is proved to be of preferred technique.[13] Many variations of thoracoscopy for obtaining pleural tissue have been devised.[14] All these methods need the sophisticated instruments and expertise; and are not readily available. In addition, there are associated risks of greater invasiveness.

The objective of the present study was to make an etiological diagnosis of pleural effusion where cytological, biochemical and microbiological examinations of pleural fluid did not help to make the diagnosis. The role of percutaneous parietal pleural needle biopsy in cases of undiagnosed exudative pleural effusion was evaluated.

## MATERIALS AND METHODS

The study was conducted in a tertiary hospital of Delhi over a period of one year. It was a non-concurrent prospective study. The academic board and ethical committee of the institution approved study protocol. Written and informed consent was sought from all participants. Patients more than 14 years of age with undiagnosed pleural effusion were the participants. The detailed clinical history and physical examination of each patient was recorded. Subsequent to the confirmation of pleural effusion by chest skiagram, diagnostic thoracentesis was performed to get the fluid for analysis. Cytological examination included total and differential leukocyte count, RBCs, mesothelial cells, malignant cells and LE cell. Biochemical tests of pleural fluid were estimation of glucose, protein, LDH and rheumatoid factor. Simultaneously blood glucose and serum proteins were also measured. The levels of ADA were also measured. The pH of pleural fluid was measured with ABG measuring equipment. Microbiological examination consisted of gram stain, smear staining, and culture for AFB. Tuberculin test was also done by using PPD. If the etiological diagnosis was confirmed by these investigations, then that patient was excluded from the study. When these investigations of pleural fluid were not able to make the diagnosis, it was labeled as undiagnosed pleural effusion and was subjected to pleural biopsy. The patients with bleeding diatheses or taking anticoagulants, borderline respiratory failure, empyema, local skin infection and non-cooperation were excluded. Twenty-five patients participated in the study. HIV status of all patients was tested.

### Pleural biopsy

The procedure of pleural biopsy was done in sitting posture of patients as described elsewhere.[15] The affected side of chest was determined and the site for biopsy was selected. This area was cleaned thoroughly with antiseptics and then infiltrated with local anesthetic (1% lignocaine). Confirmation of free fluid was acknowledged with aspiration with same syringe. For the free access, small incision of size of 0.5 cm was made just above the upper border of the rib of selected site. The Cope’s needle was introduced through it. Multiple biopsies were taken with the needle by multiple passes. After biopsy, skin incision was sutured with a single stitch. Post-biopsy chest X-ray was taken to rule out the entry of air. Pleural tissue obtained was placed in three vials; one with formalin for histopathological examination and AFB smear staining, and other two with normal saline for culture (tuberculosis and fungus). Two patients underwent thoracoscopy using rigid thoracoscope under general anesthesia for obtaining pleural tissue.

## RESULTS

The study included 25 patients of exudative pleural effusion in whom the diagnosis was not yielded by cytological, biochemical and microbiological investigations. They were 17 (68%) male and 8 (32%) female. Age of them ranged from 15 to 65 years (Mean 31.72). 60% of pleural effusions were on left side. The reports of serum ELISA for HIV was nonreactive in all of them.

Mean value of serum and pleural fluid LDH was 170.56 and 1080.28 U/L, respectively. Effusions were predominantly lymphocytic. Mean value of polymorph and lymphocyte count was 6.84% and 92.84%, respectively. In 20 out of 25 patients there were no RBCs in pleural fluid. In two patients fluid was frankly hemorrhagic. Three of 25 patients were found to have mesothelial cells in their pleural fluid: and their count was 1%, 2%, and 5%. Mean level of glucose was 64.44 mg/dl with lowest being nil; and highest level was 108 mg/dl. Mean level of protein in fluid was 5.56 gm/dl (range 3.6 to 7.20 gm/dl). Mean value of the pH of pleural fluid was 7.31 (lowest 7.066) and its higher value was 7.485. ADA was measured in 20 patients (17 patients of tuberculosis, 2 of malignancy and 1 of viral). The average values of ADA in tuberculosis, malignancy and viral were 83.82, 21.50 and 82.00 U/L, respectively. The sensitivity and specificity of ADA for detecting tubercular pleural effusion was 94.1% (cut-off value 40 U/L) and 66.7%, respectively. Tuberculin test was positive only in five patients, who were diagnosed as tubercular pleural effusion afterwards.

### Pleural biopsy

**Figure 1:**
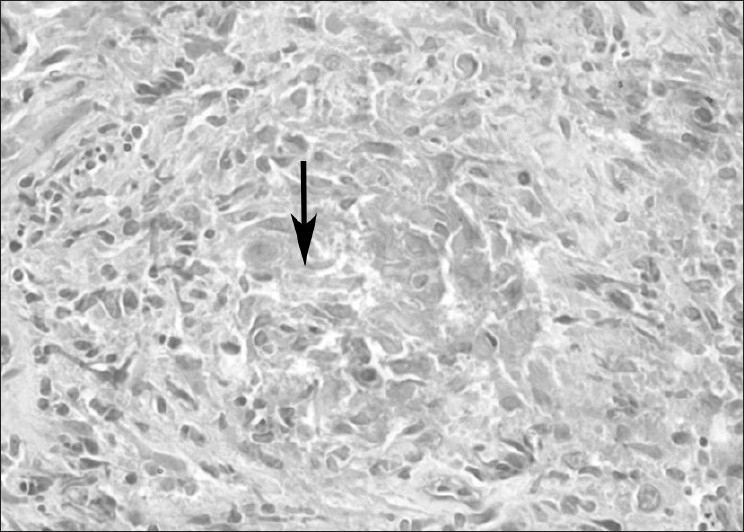
Section from pleural biopsy (arrow) showing epithelioid cell granuloma

**Figure 2:**
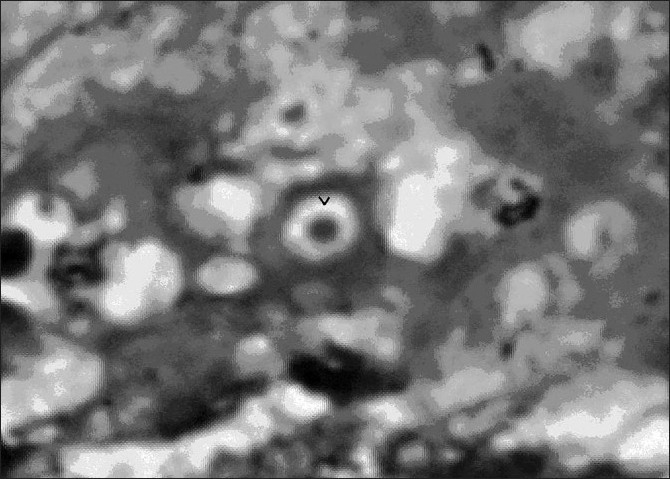
Another sample of pleural biopsy (arrow head) showing inclusion body of cytomegalovirus in center

**Figure 3:**
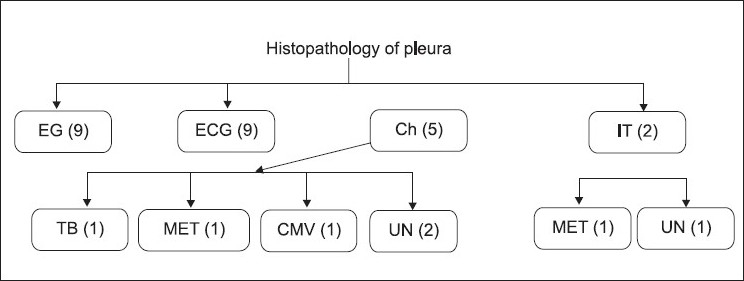
Flow diagram showing histopathology examination of pleural biopsy of 25 undiagnosed pleural effusion (EG= Epitheloid granuloma, ECG=Epitheloid granuloma with caseation, CH= Chronic Inflammation, IT= Indeterminate, TB= Tuberculosis, MET= Metastasis, CMV= Cytomegalovirus, UN= Unknown)

More than one passes were taken to obtain at least three biopsy samples. Average number of passes made were 5.08 (range 4–8) and samples obtained was 3.76 (range 3–7). Histopathology showed epitheloid granuloma with caseation necrosis [Figure 1] in 9 (36%) patients. The epitheloid granuloma with or without giant cells were reported in another 9 (36%) cases. All of them were considered as suffering from tuberculosis. In 5 (20%) patients histopathology report was of nonspecific chronic inflammation. Among them, pleural biopsy of one patient (4%) showed inclusion bodies of cytomegalovirus [Figure 2] in mesothelial cells. Another patient’s histopathology was reported as normal pleura; pleura tissue was taken by pleuroscopy and it turned out to be case of metastasis malignancy. In two (8%) patients pleural tissue obtained was inadequate for any opinion; pleural biopsy was obtained by thoracoscopy in both. One of them had metastatic malignancy and second one was turned out to be tubercular pleural effusion. Thus out of 25 patients [Figure 3], histopathology of pleural biopsy revealed tuberculosis among 19 (76%), metastasis to pleura in 2 (8%), CMV pleuritis in 1 (4%) and chronic inflammation in 3 (12%).

Pleural tissue was also submitted for Ziehl-Neelsen (ZN) staining for presence of mycobacterium tuberculosis. It was positive in 2 (10.5) of 19 patients. The histopathological report was epitheloid granuloma in one and epitheloid granuloma with caseation in other. Culture of pleural tissue for AFB was positive in 3 (15.7%) among 19 patients of tubercular pleural effusion.

Two patients were diagnosed as malignant pleural effusion (metastatic); and pleural biopsy was obtained by thoracoscopy. Incidentally, both patients had mass in one of their kidneys. The final diagnosis was renal cell carcinoma with metastasis to pleura in both of them.

Two patients out of 25 patients developed hydropneumothorax and required intercostal tube drainage. One patient developed subcutaneous emphysema at biopsy site which resolved within next two days.

## DISCUSSION

The etiology of pleural effusion remained distant despite all conventional and specific investigations. In this study an effort was made to reach an etiological diagnosis in undiagnosed exudative pleural effusion by performing percutaneous parietal pleural biopsy using Cope’s needle.

Male (68%) gender predominated in the study population. The mean age of patients was 31.72 years and 68% were younger than 34 years. The mean age of patients with tuberculous pleural effusion was 28.71 years, a little below than those reported earlier.[9] ADA was sensitive in detecting tuberculous effusion (94.1%) in present study; however, specificity was low (66.7%). ADA has been very sensitive in some studies;[16] others have noted lower results.[17–19] Estimation of alkaline phosphatase activity in pleural fluid was demonstrated a useful test in differentiation of tuberculous from nontuberculous pleural effusion;[20] however, this test was not done in this study. Tuberculin test was positive in 5 (25%) patients of proved tubercular effusion in the present study. Tuberculin test was negative among 30% of patients with tuberculous pleural effusion; this test may be non-reactive initially but after 6 to 8 weeks of observation may convert to reactive.[21]

Adequate pleura was obtained in 23 (92%) of cases in this study almost similar to others.[3] Nineteen (76%) of 23 patients (in whom adequate pleura was obtained) were diagnosed as a definite disease by histopathology. Thus the diagnostic yield of Cope’s needle was midway between earlier reports.[17,22,23]

The diagnosis of tuberculosis based on histopathology reports as epithelioid granuloma with or without giant cells and necrosis was made among 18 patients. Almost same results have been reported previously.[17] In two patients, AFBs were seen in histological section of pleura stained by Ziehl-Neelsen staining. While one series had not seen AFB in tissue smears[22] the other had reported comparatively higher percentage.[19] Culture for Mycobacterium tuberculosis was positive in only 12% in this study, which is quite low in comparison to others.[14,17] It is assumed that the small tissue obtained by Cope’s needle, single sample sent for culture and AFB staining, and the bias of investigator of smaller tissue sample for culture besides the other usual causes like the meticulousness shown by the microbiologist for culturing the sample might be the reasons for low positivity for AFB in present series. However, visualization or positive culture of AFB did not contribute additionally for making the diagnosis because they were diagnosed based on histopathology. Diagnostic yields of pleural biopsies in tuberculosis had been 20%.[24] Other factors like bulk of pleural tissue obtained[15] and the number of times biopsy performed[24] also determined the success rate.

In five patients the histopathology report was chronic inflammation; and in one patient it was normal pleura. Nonspecific inflammation with varying degrees of fibrosis or normal pleura was found in 68% of patients in one study.[25] One patient’s histopathology showed cytomegalovirus (CMV) inclusion bodies inside the mesothelial cells. Viruses are presumed to be one of the common causes of undiagnosed exudative pleural effusion but histological documentation is not always there.[26] Unlike this patient, in most of the reports patients were immunocompromised.[27,28]

Significance of needle biopsy in malignant pleural effusion cannot be accurately estimated from this study as only two patients were with malignant effusion. Both patients ultimately turned out to be having renal cell carcinoma with metastasis to pleura.

In conclusion, the role of percutaneous closed needle biopsy of pleura in cases of undiagnosed exudative pleural effusion is still pivotal as it reached a specific diagnosis in majority of cases. This is of particular importance in a developing country like India where the facilities of thoracoscopy and imaging guided cutting needle biopsies are not easily available. In addition, needle biopsy causes little morbidity and no mortality. This can be performed with little instrumental and manpower support.
